# Crude venom from nematocysts of *Pelagia noctiluca* (Cnidaria: Scyphozoa) elicits a sodium conductance in the plasma membrane of mammalian cells

**DOI:** 10.1038/srep41065

**Published:** 2017-01-23

**Authors:** Rossana Morabito, Roberta Costa, Valentina Rizzo, Alessia Remigante, Charity Nofziger, Giuseppa La Spada, Angela Marino, Markus Paulmichl, Silvia Dossena

**Affiliations:** 1 University of Messina, Department of Chemical, Biological, Pharmaceutical and Environmental Sciences, Viale Ferdinando Stagno D’ Alcontres 31, Messina, I-98166, Italy; 2 Paracelsus Medical University, Institute of Pharmacology and Toxicology, Strubergasse 21, Salzburg, A-5020, Austria

## Abstract

Cnidarians may negatively impact human activities and public health but concomitantly their venom represents a rich source of bioactive substances. *Pelagia noctiluca* is the most venomous and abundant jellyfish of the Mediterranean Sea and possesses a venom with hemolytic and cytolytic activity for which the mechanism is largely unknown. Here we show that exposure of mammalian cells to crude venom from the nematocysts of *P. noctiluca* profoundly alters the ion conductance of the plasma membrane, therefore affecting homeostatic functions such as the regulation and maintenance of cellular volume. Venom-treated cells exhibited a large, inwardly rectifying current mainly due to permeation of Na^+^ and Cl^−^, sensitive to amiloride and completely abrogated following harsh thermal treatment of crude venom extract. Curiously, the plasma membrane conductance of Ca^2+^ and K^+^ was not affected. Current-inducing activity was also observed following delivery of venom to the cytosolic side of the plasma membrane, consistent with a pore-forming mechanism. Venom-induced NaCl influx followed by water and consequent cell swelling most likely underlie the hemolytic and cytolytic activity of *P. noctiluca* venom. The present study underscores unique properties of *P. noctiluca* venom and provides essential information for a possible use of its active compounds and treatment of envenomation.

Cnidarians envenomation may result of concern for public health and represent a medical urgency. Consequences of Cnidaria stings may range from minor local irritation to severe local and systemic reactions including excruciating pain and life-threatening cardiovascular collapse depending on the Cnidarian species, age of the victim and size of the area contacted. In addition, Cnidarians outbreaks may interfere with human activities such as tourism, bathing, aquatic events, fishing and aquaculture, causing substantial economical burden^1^,^2^,^3^,^4^. On the other hand, Cnidarians venom is a rich source of bioactive substances that may have therapeutic potential and other useful applications^5^,^6^,^7^. Owing to their activity on voltage-gated ion channels in the central nervous system and transmitter release at the neuromuscular junction, Cnidaria toxins can substantially contribute to the development of lead compounds for the treatment of pain and some neurological and neurodegenerative diseases^6^,^7^. Cytotoxic and cytolytic properties of many Cnidaria venoms are specific for distinct cell types, therefore suggesting potential use as anti-cancer^5^, antibiotic, antiviral and antiparasitic agents^8^. Not limited to human medicine, further possible applications of Cnidaria toxins include its use as insecticides, acaricides and antifouling agents^9^,^10^,^11^. Treatment of jellyfish stings, prevention of possible harmful local and systemic reactions consequence of the sting and exploitation of the bioactive substances contained in the venom require a precise knowledge of the mechanism of action of venom compounds.

*Pelagia noctiluca* (Forsskål, 1775), commonly called purple-striped jelly or mauve stinger, is a bioluminescent jellyfish with wide distribution in coastal warm and temperate waters, including the Pacific and Atlantic Oceans, and particularly abundant in the Mediterranean Sea, where it is considered the most venomous autochthonous jellyfish^12^. Similarly to all Cnidarians, *P. noctiluca* possesses stinging cells, the nematocytes, which comprise a specialized organoid, the nematocyst, having the function of discharging into the teguments of a prey or predator a complex mixture of toxic substances contained within the capsular fluid. Accidental contact with *P. noctiluca* specimens may cause painful local and severe generalized symptoms in humans, including erythema, edema, vesicular topical lesions, persistent scaring and hyperpigmentation, cutaneous eruptions, and allergic reactions and cross-reactions, which are particularly harmful in sensitive subjects^13^. During the last decades, abnormal proliferation of this jellyfish in the Mediterranean basin represented a remarkable threat to the public health and caused substantial economical burden by interfering with human activities such as tourism and fishery, consequently arousing increasing interest on the toxicological properties of its venom^12^.

The biological activity of crude venom extracted from nematocysts of *P. noctiluca*, and therefore not including other tissue-derived compounds, has been tested both *in vitro* and *in vivo*^14^. Crude venom induced time and dose-dependent erythrocyte lysis, putatively *via* cell membrane damage, with toxic activity affected by osmotic protectants, divalent cations and heavy metals^15^,^16^,^17^,^18^. Toxicity was attributed to the activity of a protein constituent likely recognizing specific targets in the plasmatic membrane of red blood cells^19^. *P. noctiluca* crude venom also showed remarkable cytotoxic properties on cultured cells^20^,^21^,^22^. Specifically, anti-proliferative activity, integrin-dependent inhibition of cell adhesion to the extracellular matrix, reactive oxygen species (ROS) production and mitochondrial transmembrane potential collapse have been shown in glioblastoma^23^ and neuronal-like (SH-SY5Y) cells^24^, respectively. Following injection in the rat paw, *P. noctiluca* crude venom elicited an acute inflammatory response, including local edema, polymorphonuclear neutrophil infiltration, lipid peroxidation, nitrosative stress and cell apoptosis in the paw tissue^25^. Intravenous injection of crude venom in rats evoked a systemic inflammatory response along with increased plasma levels of nitric oxide and ROS as well as cellular infiltration, increased cyclooxygenase expression, lipid peroxidation and induction of apoptosis in the lung and intestine^26^.

The mechanism underlying the hemolytic and cytolytic properties of venoms of Cnidaria, including *P. noctiluca*, is only partially understood and may rely on alterations of ion and water fluxes through the plasma membrane of target cells. According to this hypothesis, nematocytes isolated from the Anthozoan *Aiptasia mutabilis*, exposed to *P. noctiluca* crude venom and submitted to hypotonic stress failed to regulate their volume. This effect was dose-dependent and reversed by the K^+^ ionophore gramicidin^27^. The last observation suggested an inhibitory effect of *P. noctiluca* venom on cell membrane ion transport mechanisms involved in the process of regulatory volume decrease, which relies on K^+^ efflux in most cells.

The regulation of cellular volume is an essential homeostatic function of mammalian cells in which cell morphological integrity and function is preserved despite osmotic imbalances between the intracellular and extracellular fluid. In a variety of physiological and pathological conditions, a reduction in the osmolarity of the extracellular milieu or an increase in the osmolarity of the cytosol may lead to an obligate, osmotic-driven water influx and cell swelling. The phenomenon by which swollen cells reduce their volume towards resting values to prevent cell lysis is called regulatory volume decrease (RVD). During RVD, the activation of electroneutral and electrogenic ion transport mechanisms for the export of K^+^ and Cl^−^ ions and other osmolytes generates an obligate efflux of water, and consequently leads to a reduction of cellular volume. The activation of a Cl^−^ conductance (IClswell) is an essential step during RVD and is seen in most cells^28^,^29^,^30^.

Cell swelling may represent the initial step towards cell lysis following exposure to Cnidaria venom. In the present investigation, we verified if crude venom isolated from the nematocysts of *P. noctiluca* could affect the regulation of cellular volume and membrane ion transport pathways in mammalian cells. We observed that *P. noctiluca* crude venom profoundly altered RVD and caused dramatic cell swelling even in the absence of osmotic gradients by eliciting a Na^+^ conductance.

## Results

### *P. noctiluca* crude venom profoundly affects the regulatory volume decrease (RVD) in human cells and induces cell swelling in the absence of osmotic gradients

**Figure 1:**
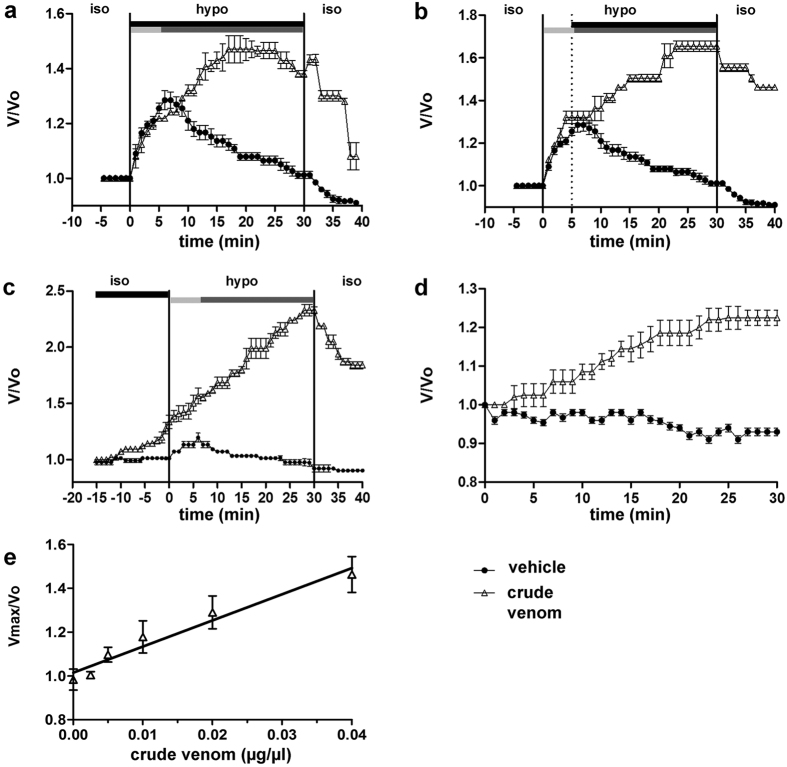
Crude venom affects the regulation and maintenance of volume in HEK 293 Phoenix cells. Cell volume, as V/V_0_ or Vmax/V_0_, is plotted against time or venom concentration. (**a–c**) Vehicle-treated cells exposed to a hypotonic challenge exhibited the expected swelling (osmotic phase, light grey bars), reaching a V/V_0_ peak value after a 6 min exposure to the hypotonic solution, and subsequently regulated their volume (RVD phase, dark grey bars) towards the value initially observed in isotonic solution. Addition of 0.025 μg/μl crude venom to the hypotonic solution (either from the beginning, (**a**) or after a 5 min exposure to the hypotonic solution, (**b**) dotted line) led to dramatic cell swelling and completely prevented RVD. (**c)** pre-incubation of cells for 15 min in isotonic solution containing crude venom lead to dramatic cell swelling and abrogated RVD during a subsequent exposure to a hypotonic solution not containing crude venom. (**d)** Exposure of cells to an isotonic solution containing crude venom led to significant cell swelling. After 30 min, V/V_0_ for vehicle and venom-treated cells was respectively 0.93 ± 0.01, n = 4, and 1.23 ± 0.02, n = 4, p < 0.0001, unpaired Student’s t test. (**e**) Cells exposed to increasing concentrations of crude venom exhibited dose-dependent volume changes; n = 6–12 for each data point. Black bars in (**a,b** and **c**) indicate the presence of crude venom or the vehicle in the experimental solutions. Iso, isotonic solution 1 (Table 1); hypo, hypotonic solution 2 (Table 1). Refer to Supplementary Table 1 for statistics of panels a, b and c.

As previously mentioned, crude venom of *P. noctiluca* alters the osmotic response of *A. mutabilis* nematocytes to an extracellular hypotonic solution by preventing RVD^27^. A similar phenomenon may likely occur in human cells. To verify this hypothesis, RVD was monitored in HEK 293 Phoenix cells exposed to isotonic (solution 1, Table 1) or hypotonic (solution 2, Table 1) solutions containing either *P. noctiluca* crude venom (0.025 μg/μl protein) or the vehicle. Cells, initially kept in isotonic solution, were exposed to a hypotonic solution containing the crude venom or vehicle, and then returned to an isotonic solution (Fig. 1a). In the presence of the vehicle, cells initially swelled (osmotic phase) and successively regulated their volume towards the initial value (RVD phase). In the presence of a venom-containing hypotonic solution, the RVD phase was completely abrogated (Fig. 1a). Similar results were observed when the crude venom was added to the hypotonic solution after the osmotic phase (*i.e.* after a 5 min exposure of cells to an extracellular venom-free hypotonic solution, Fig. 1b). Similarly, preincubation of cells for 15 min in an isotonic solution containing 0.025 μg/μl crude venom completely abrogated RVD in a venom-free hypotonic solution, evidencing the irreversibility of venom-induced derangements (Fig. 1c).

Most interestingly, exposure of cells for 30 min to an isotonic solution containing 0.025 μg/μl crude venom caused a significant increase in volume that was not observed in cells exposed to the vehicle (Fig. 1d). The venom-induced cell swelling in isotonic solution was dose-dependent, and exhibited a linear (r^2^ = 0.96) dose-response relationship (Fig. 1e).

### Venom-induced regulatory volume decrease (RVD) impairment is not derived from inhibition of swelling-dependent Cl^−^ channels

**Figure 2:**
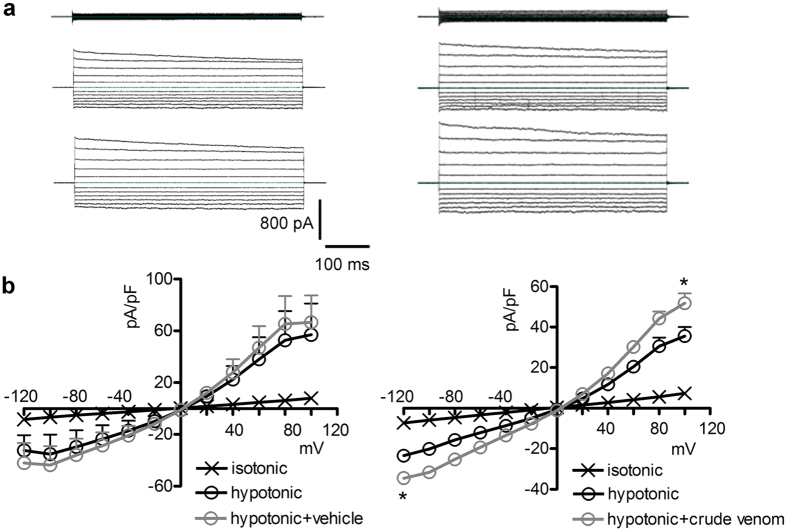
Crude venom does not inhibit IClswell. (**a**) Original current recordings obtained from HEK 293 Phoenix cells in whole-cell configuration with pipette solution 1 (Table 2) and bath solutions 1 (isotonic, Table 1) – top panels – and 2 (hypotonic, Table 1) – middle and lower panels – before and after addition of the vehicle (left panels) or crude venom (right panels) to the hypotonic bath solution. (**b**) Current density (pA/pF) to voltage (mV) relationship of Cl^−^ currents obtained in isotonic and hypotonic solutions before and after the addition of the vehicle (left; n = 5) or 0.025 μg/μl crude venom (right; n = 4) to the hypotonic solution after complete activation of IClswell (*i.e.* after a ~20 min exposure to the hypotonic solution). *p < 0.05 compared to hypotonic, paired Student’s t test.

**Figure 3:**
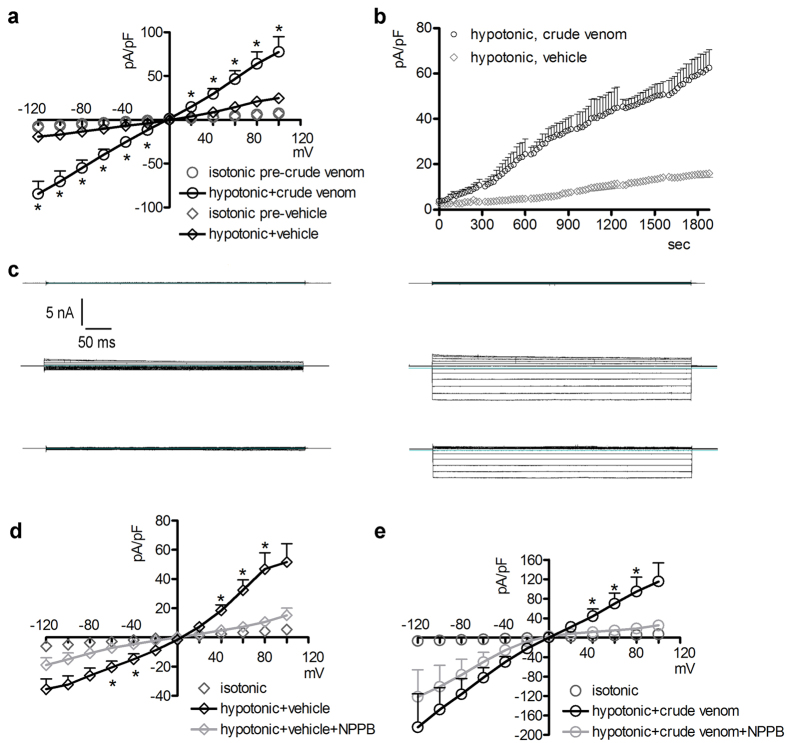
Crude venom greatly increases the magnitude of hypotonicity-stimulated ion currents. (**a**) Current density (pA/pF) to voltage (mV) and (**b**) current density (pA/pF) to time (sec) relationship of currents obtained from HEK 293 Phoenix cells in whole-cell configuration with pipette solution 1 (Table 2) and bath solutions 1 (isotonic, Table 1) and 2 (hypotonic, Table 1). The hypotonic solution contained either the vehicle (n = 42) or 0.025 μg/μl crude venom (n = 26). The current density-to-voltage relationship represented in (**a**) was measured before and after a 15 min hypotonic challenge. *p < 0.001 compared to hypotonic +vehicle, unpaired Student’s t test. In (**b**) p < 0.05 for all time points. (**c**) Original current recordings obtained with the above mentioned pipette and isotonic (top panels) and hypotonic bath solutions (middle panels) and after the addition of 60 μM NPPB to the hypotonic bath solution (lower panels) in vehicle (left) or venom-treated (right) cells. Current density-to-voltage relationship obtained with the abovementioned pipette, isotonic and hypotonic solutions (after a 30 min hypotonic challenge) containing either the vehicle (n = 6), (**d**) or crude venom (n = 6), (**e)** and after the addition of NPPB to the hypotonic solution. *p < 0.05 compared to hypotonic +NPPB, paired Student’s t test.

As previously mentioned, swelling-dependent Cl^−^ or K^+^ channels are major determinants of RVD in most cells, and lack or impairment of RVD may arise from inhibition of these conductances^29^. In addition, volume-sensitive chloride channels are involved in maintenance of basal cellular volume in some cell types^31^. Therefore, we verified if venom-induced RVD impairment and cell swelling in isotonic conditions could be the consequence of an inhibition of swelling-dependent Cl^−^ or K^+^ channels. Electrophysiological measurements were performed by whole-cell patch-clamp on HEK 293 Phoenix cells with bath and pipette-filling solutions suitable for measuring the swelling-activated chloride current IClswell (solutions 1 and 2, Table 1, and solution 1, Table 2). The seal was realized and the whole cell configuration was obtained with cells initially kept in extracellular isotonic solution. In this condition, no obvious chloride currents were detected (Fig. 2a, top panels). IClswell activation was elicited following reduction of the extracellular osmolarity of 50 mOsm/Kg_H2O_ *via* omission of mannitol. Hypotonic shock induced the activation of a large chloride current with the biophysical fingerprints of IClswell (outward rectification, slow voltage- and time-dependent inactivation at potentials higher than +40 mV; Fig. 2a, middle panels), similar to previous observations^32^,^33^,^34^. The reversal potential of these currents is very close to 0 mV, *i.e.* the equilibrium potential predicted for chloride with the experimental solutions used, indicating the chloride selectivity of the elicited conductance (Fig. 2b). Addition of 0.025 μg/μl crude venom to the hypotonic solution after complete activation of IClswell did not inhibit, but rather led to a further increase of the current that was not observed in cells treated with the vehicle (Fig. 2b). To further explore this effect, IClswell activation was elicited in the presence of 0.025 μg/μl crude venom or the vehicle. The current magnitude measured in cells treated for 15 min with a hypotonic solution containing the crude venom was significantly larger than that observed in cells exposed to the vehicle (Fig. 3a,b). The biophysical and pharmacological fingerprints of IClswell induced in the presence of venom appeared distinct from those of the endogenous IClswell. The current-to voltage relationship was linear rather than outwardly rectifying (Fig. 3a and c), the activation kinetic was significantly faster (Fig. 3b) and the inward component was relatively insensitive to the chloride channel blocker 5-nitro-2-(3-phenylpropyl-amino) benzoic acid (NPPB) (Fig. 3c,d and e). These data suggest that the venom-induced impairment of RVD is not related to an inhibition of a Cl^−^ conductance.

### Venom-induced regulatory volume decrease (RVD) impairment is not derived from inhibition of K^+^ channels

**Figure 4:**
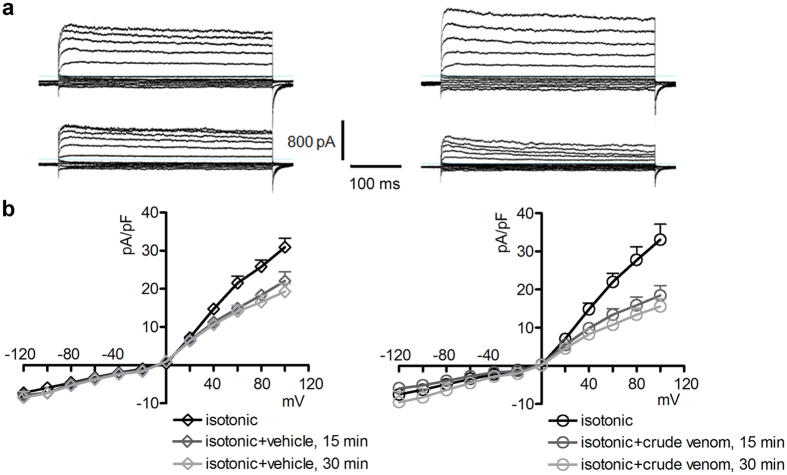
Crude venom does not modify the K^+^ conductance of HEK 293 Phoenix cells. (**a**) Original current recordings obtained in whole-cell configuration with pipette solution 2 (Table 2) and bath solution 3 (Table 1) before (top panels) and 15 min after (lower panels) addition of the vehicle (left panels) or 0.025 μg/μl crude venom (right panels) to the bath solution. (**b**) current density (pA/pF) to voltage (mV) relationship of K^+^ currents before, 15 and 30 min after addition of the vehicle (left; n = 14) or crude venom (right; n = 7) to the hypotonic solution. No significant differences in current intensity between vehicle or venom-treated cells were detected, unpaired Student’s t test.

HEK 293 Phoenix cells express a voltage-dependent K^+^ -selective current exhibiting a spontaneous rundown over time after establishing the whole-cell configuration^35^. Rather than activating these currents, hypotonicity seems to slow their rundown (see Supplementary Fig. S1). Crude venom (0.025 μg/μl), added to an extracellular K^+^ -rich isotonic solution (solution 3, Table 1) for 30 min neither inhibited or modified the biophysical properties of the voltage-dependent K^+^ -selective current (Fig. 4), suggesting that the venom-induced impairment of RVD is not related to an inhibition of a K^+^ conductance.

### *P. noctiluca* crude venom increases the membrane permeability to ions

**Figure 5:**
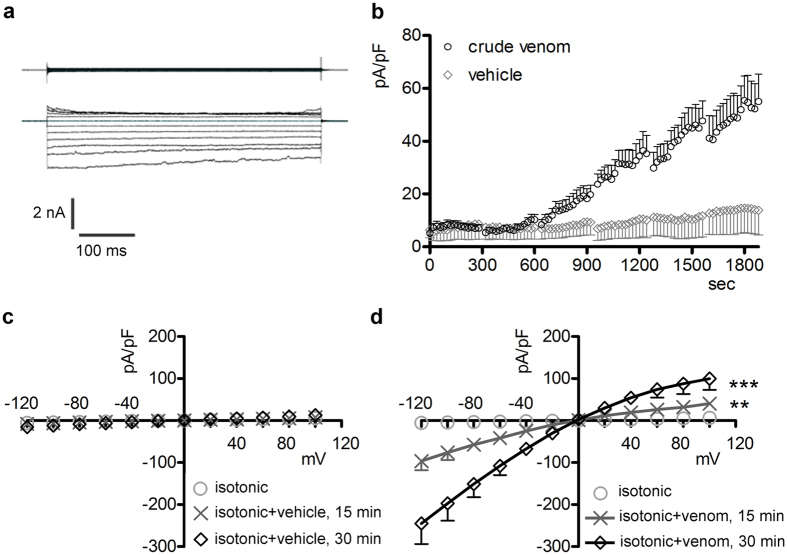
Crude venom elicits an inwardly-rectifying current. (**a**) Original current recordings obtained from HEK 293 Phoenix cells in whole-cell configuration with pipette solution 1 (Table 2) and bath solution 1 (isotonic, Table 1), before (top panel) and 30 min after addition of 0.025 μg/μl crude venom to the bath solution (lower panel). (**b**) Current density (pA/pF) to time (sec) and (**c,d**) current density (pA/pF) to voltage (mV) relationship recorded in isotonic solution before, 15 and 30 min after the addition of the vehicle (left; n = 11) or crude venom (right; n = 12) to the bath solution. **, ***p < 0.01, p < 0.001 compared to vehicle at all applied voltages except 0 mV, unpaired Student’s t test. In (**b**) p < 0.05 for time points > 900 sec.

**Figure 6:**
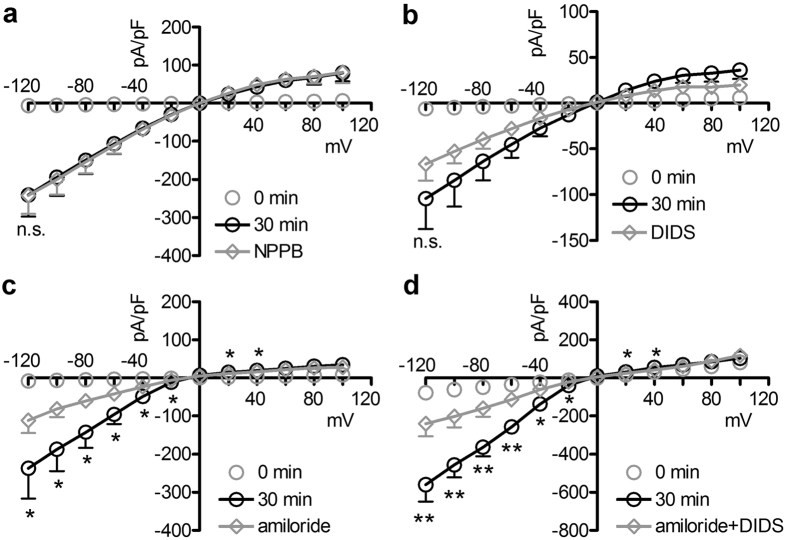
The venom-induced current is inhibited by amiloride. (**a–c**) Current density (pA/pF) to voltage (mV) relationships obtained from HEK 293 Phoenix cells in whole-cell configuration with pipette solution 1 (Table 2) and bath solution 1 (isotonic, Table 1), before (0 min) and after (30 min) the addition of 0.025 μg/μl crude venom to the bath solution. Cells were subsequently exposed to an isotonic solution containing crude venom and 60 μM of (**a**) NPPB (n = 5), (**b**) DIDS (n = 5), (**c**) amiloride (n = 6), (**d**) amiloride plus DIDS (n = 4). n.s., not significant; *, **p < 0.05, p < 0.01 compared to amiloride or amiloride plus DIDS, paired Student’s t test.

The venom-induced modifications of the biophysical properties of IClswell (Figs 2 and 3) suggest that the crude venom may be *per se* capable of inducing currents. To verify this hypothesis, whole-cell currents were recorded in HEK 293 Phoenix cells exposed to an extracellular NaCl-rich isotonic solution (solution 1, Table 1) containing either 0.025 μg/μl crude venom or the vehicle. In cells exposed for 30 min to the vehicle, no obvious currents were detected. In contrast, in cells exposed to the venom, a large inwardly rectifying current that increased over time appeared (Fig. 5). The venom-induced current was resistant to the chloride channel inhibitor NPPB (Fig. 6a) and poorly sensitive to 4,4′-Diisothiocyano-2,2′-stilbenedisulfonic acid (DIDS, Fig. 6b), but was efficiently blocked by the epithelial Na^+^ channel inhibitor amiloride (current inhibition at −120 mV: 46 ± 12%, n = 6, Fig. 6c). The combination of DIDS and amiloride (Fig. 6d) further inhibited the venom-induced current (current inhibition at −120 mV: 71 ± 9%, n = 4). The vehicle (0.6–1.2% dimethyl sulfoxide) did not significantly modify the venom-induced current (data not shown). Crude venom induced a current with similar characteristics in HeLa cells and mouse NIH/3T3 fibroblasts (see Supplementary Fig. S2).

### The predominant component of the venom-induced current is not a chloride conductance

To establish the ion selectivity of the venom-induced current, Cl^−^ and/or Na^+^ in the extracellular solution of venom-exposed cells were substituted with impermeant ions and the reversal potential (Erev) of the current was determined. First, the venom-induced current was elicited in an isotonic, NaCl-rich solution (solution 1, Table 1). Then, the Cl^−^ concentration in the extracellular solution was reduced (solution 5, Table 1; complete omission of Cl^−^ in a patch-clamp set-up equipped with Ag/AgCl electrodes is inapplicable). This maneuver induced a shift of Erev from −14.5 ± 3.5 to −2.5 ± 2.4 mV (p < 0.02, n = 5), that, although statistically significant, did not reach the Erev predicted by the Nernst law for a chloride-selective current (that is, +50 mV). These results indicate that the main component of the venom-induced current is not the consequence of permeation of Cl^−^, although a small Cl^−^-dependent component may also be present.

### The predominant component of the venom-induced current is a sodium conductance

**Figure 7:**
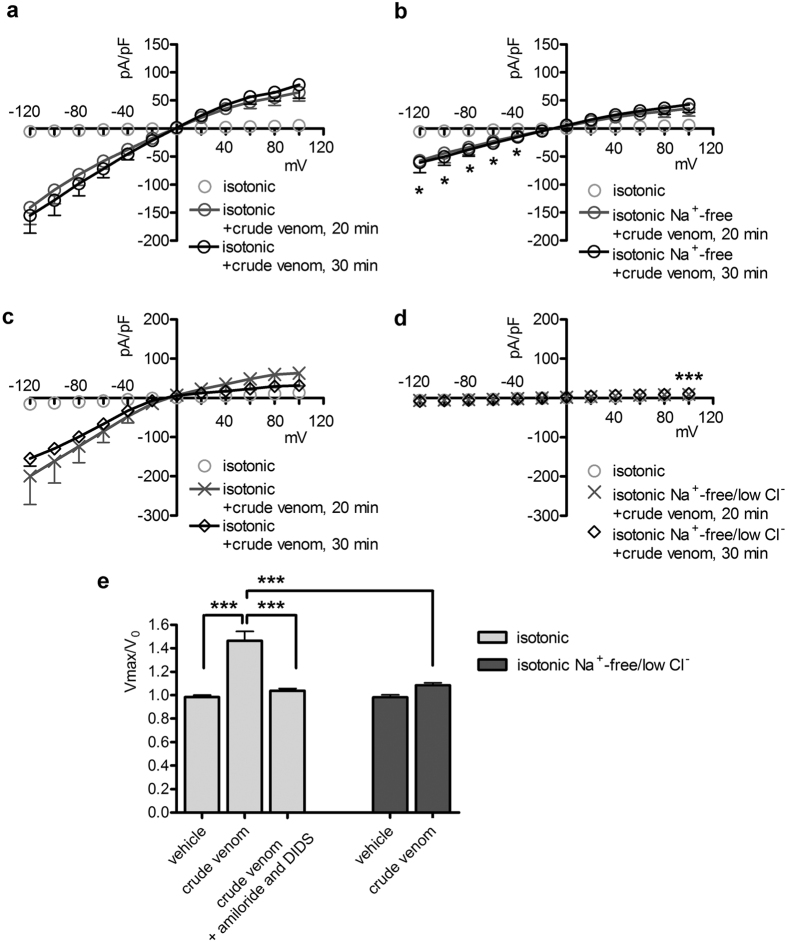
Crude venom greatly increases the membrane conductance for sodium. (**a,c**) Current density (pA/pF) to voltage (mV) relationship obtained from HEK 293 Phoenix cells in whole-cell configuration with pipette solution 1 (Table 2) and bath solution 1 (isotonic, Table 1), before, 20 and 30 min after the addition of 0.025 μg/μl crude venom to the bath solution. The same batch of venom as in (**a**) pipette solution 1 (Table 2) and isotonic bath solution 1 (Table 1) were also used for the experiments represented in (**b**). In (**b**) cells were subsequently exposed to crude venom in an isotonic solution not containing Na^+^ (solution 6, Table 1). *p < 0.05 compared to (**a**) isotonic +crude venom, 20 min, unpaired Student’s t test. n was 18 in (**a)** and 20 in (**b**) respectively. The same batch of venom as in (**c**) pipette solution 3 (Table 2) and isotonic bath solution 1 (Table 1) were used for the experiments represented in (**d**). In (**d**) cells were subsequently exposed to crude venom in an isotonic solution not containing Na^+^ and with a reduced concentration of Cl^−^ (solution 7, Table 1). ***p < 0.001 at all applied voltages except 0 mV compared to (**c)** isotonic +crude venom, 30 min, unpaired Student’s t test; n = 6 for (**c** and **d**). (**e**) Cells were kept in isotonic (solution 1, Table 1) or isotonic Na^+^ -free/low Cl^−^ (solution 7, Table 1) bath solution in the presence of crude venom, amiloride and DIDS or the respective vehicles and the maximal volume changes (Vmax/V_0_) measured. n = 6 for each condition. ***p < 0.001, one-way ANOVA with Bonferroni’s post-hoc test.

To identify the main permeant ion determining the venom-induced current, Na^+^ was completely removed from the extracellular solution (solution 6, Table 1). In these conditions, the current density in venom-exposed cells, although not annihilated, was significantly reduced (Fig. 7a,b). These results indicate that the main component of the venom-induced current is the consequence of a permeation of Na^+^ and further confirm that a small Cl^−^-dependent component is also present. Accordingly, removal of Na^+^ and reduction of Cl^−^ concentration in the extracellular (solution 7, Table 1) and intracellular solutions (solution 3, Table 2) completely abrogated the ability of the venom to increase the cell membrane permeability to ions (Fig. 7c,d). Indeed, currents shown in Fig. 7d were not statistically different from those measured in a set of control experiments obtained with pipette solution 1 (Table 2), bath solution 1 (isotonic, Table 1) and in the presence of the vehicle (n = 11; not shown).

### Conditions reducing the venom-induced current also impede the venom-induced cell swelling

As detailed above, the venom-induced current was substantially reduced or completely abrogated by (i) exposure of venom-treated cells to a combination of DIDS and amiloride or (ii) removal of Na^+^ and reduction of Cl^−^ in the extracellular medium. The increase of cellular volume observed in a venom-containing isotonic solution (solution 1, Table 1) was completely prevented by the inhibitors of the venom-induced current (DIDS and amiloride, Fig. 7e). Also, bathing of cells in isotonic solution containing a low concentration of Cl^−^ and no Na^+^ (solution 7, Table 1) significantly blunted the ability of crude venom to increase the cellular volume (Fig. 7e).

### The current-inducing component of *P. noctiluca* crude venom is thermosensitive

**Figure 8:**
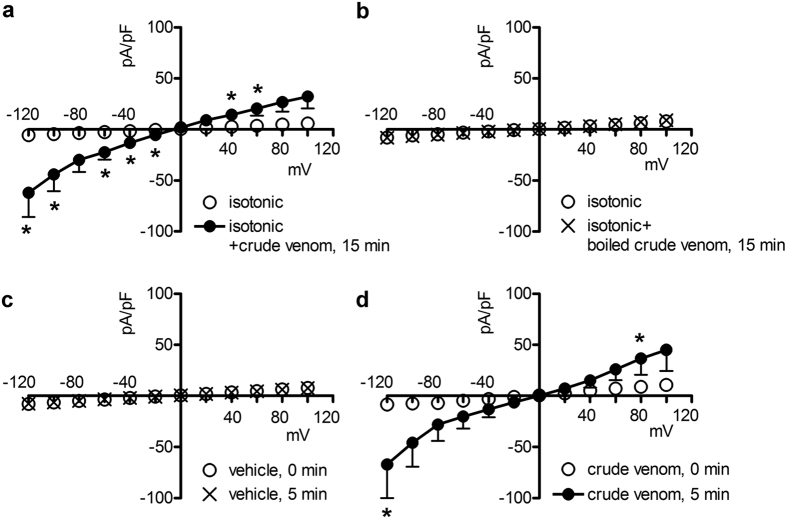
The current-inducing activity of crude venom is compatible with the characteristics of a pore-forming protein. Current density (pA/pF) to voltage (mV) relationship obtained from HEK 293 Phoenix cells in whole-cell configuration with pipette solution 1 (Table 2) and bath solution 1 (isotonic, Table 1), before and 15 min after the addition of (**a**) 0.025 μg/μl untreated (n = 7), or (**b**) boiled (n = 7) crude venom to the bath solution. *p < 0.05 compared to boiled crude venom, unpaired Student’s t test. Current density-to-voltage relationship obtained from HEK 293 Phoenix cells in whole-cell configuration with bath solution 1 (isotonic, Table 1) and pipette solution 1 (Table 2) containing either the vehicle (n = 5), (**c)** or 0.025 μg/μl crude venom (n = 4), (**d**) *p < 0.05 compared to the vehicle, unpaired Student’s t test.

Whole-cell currents were recorded in HEK 293 Phoenix cells exposed to an extracellular NaCl-rich isotonic solution (solution 1, Table 1) containing either untreated or boiled 0.025 μg/μl crude venom. In cells exposed for 15 min to raw venom, a large inwardly rectifying current appeared (Fig. 8a). In contrast, no obvious currents were detected in cells exposed to boiled venom (Fig. 8b), indicating that the current-inducing component is inactivated following exposure to high temperature, compatible with the characteristics of a protein.

### The current-inducing activity of *P. noctiluca* crude venom is compatible with a pore-forming mechanism

The experiments illustrated above show that adding the crude venom to the extracellular (bath) solution of mammalian cells leads to the activation of a large current mainly due to the permeation of Na^+^ ions. This phenomenon may be the consequence of direct insertion of a pore-forming constituent of venom into the plasma membrane, or activation of Na^+^ channels endogenously expressed in cells *via* binding to an extracellular channel domain or activating receptor. To discriminate between these two mechanisms, crude venom was added to the intracellular pipette filling solution (solution 1, Table 2) that, after establishing the whole-cell configuration, accesses the intracellular milieu. Crude venom was capable of inducing currents also in this latter condition (Fig. 8c,d), indicating that binding to an extracellular structure is not an essential prerequisite of venom-dependent current-inducing mechanism.

## Discussion

The complex composition of Cnidaria venom was at least partially determined for some species, and includes non-protein and protein components. The latter category comprises enzymes, neurotoxins and pore-forming toxins with cytolytic and hemolytic activity^36^,^37^. Pore-forming toxins (porins) are further divided in the two broad groups of actinoporins, found in Anthozoa and Hydrozoa, and jellyfish toxins. In this latter category, cubozoan-related porins, originally reported in Cubozoa species, including the deadly *Chironex fleckeri*^38^,^39^, were later reported to also be produced by Hydrozoa, Anthozoa and Scyphozoa^36^,^40^. Compared to Anthozoa, the properties of the venom of Scyphozoa have been less extensively studied. Crude or fractionated venom from cnidocysts or extracts from the whole animal, tentacles or oral arms variably affected cell morphology, proliferation, survival and adhesion with cytotoxic effects linked to cell membrane lysis, proteolysis and oxidative damage^5^. Hemolytic and cytolytic properties of Scyphozoa venom has been attributed to plasma membrane damage consequence of lipid peroxidation and pore-formation^41^. The presence of pore-forming toxins within jellyfish venom has been postulated following observations of venom-induced increases in Ca^2+^ and Na^+^ influx and K^+^ efflux across the cell membrane *via* non-selective cation channels^41^,^42^,^43^,^44^ and evidenced by transmission electron microscopy^43^,^45^ or proteomic analysis^46^.

This is the first study exploring in detail the ability of crude venom from the nematocysts of the Schypozoan *P. noctiluca* to modify the electrophysiological properties of mammalian cells. Alterations in the activity of endogenous channels can profoundly affect cell homeostasis, including the regulation of cellular volume. In fact, *P. noctiluca* crude venom prevented RVD (Fig. 1a–c) and caused significant cell swelling regardless of the presence of osmotic gradients (Fig. 1d,e) in cultured human kidney cells, a phenomenon not related to inhibition of swelling-dependent Cl^−^ and K^+^ channels endogenously expressed in these cells (Figs 2 and 4). Indeed, the current recorded in the presence of crude venom during cell swelling was increased and showed biophysical and pharmacological properties substantially different from the endogenous IClswell (Fig. 3), suggesting that crude venom can elicit an ion conductance. Therefore, the possible ability of venom to induce ion currents was verified in three distinct (two epithelial and one fibroblast) cell lines, in conditions where the basal membrane currents were undetectable in the whole-cell configuration of patch clamp, *i.e.* in the absence of K^+^ and osmotic gradients across the plasma membrane. In these conditions, exposure of cells to crude venom invariably led to the activation of a large inward-rectifying current resistant to Cl^−^ channel inhibitors and sensitive to amiloride (Figs 5 and 6 and Supplementary Fig. S2). Removal of Na^+^ from the experimental solutions substantially reduced but did not completely annihilate the venom-induced current (Fig. 7). In the presence of a reduced concentration of Cl^−^ and absence of Na^+^, exposure of cells to crude venom failed to elicit any detectable current, evidencing that the venom-induced current is represented by a predominant component due to permeation of Na^+^, as well as a Cl^−^ component (Fig. 7).

Jellyfish venoms were also reported to affect permeation of K^+^ and Ca^2+^ 41,42,43,44. In the present study, *P. noctiluca* crude venom neither modified the activity of voltage-dependent K^+^ channels endogenously expressed in HEK 293 Phoenix cells, nor elicited K^+^ currents (Fig. 4). In addition, in Na^+^ -free, low Cl^−^ solutions containing physiological concentrations of Ca^2+^ and Mg^2+^, no ion current was detected (Fig. 7), showing that the venom-induced ion conductance is not permeable to divalent cations. The ion selectivity properties of the venom-induced current are consistent with the activity of a pore-forming component and, as observed for other channel types, may rely on biophysical features of the ion permeation pore, such as diameter and exposed charges^47^,^48^, and further lead to exclude that the venom-induced ion permeation relies on a generic membrane damage due to unspecific disruption of lipid bilayer.

As detailed above, Cnidaria venom components can variably affect the ion conductance of the plasma membrane of target cells by altering the activity of resident ion channels or forming new ion permeation pathways following direct insertion of pore-forming proteins in the lipid bilayer. Specifically referred to Schypozoa, both mechanisms have been postulated^15^,^41^,^44^. In the present study, the venom-induced Na^+^ current may be the consequence of activation of dormant, endogenous Na^+^ channels or due to Na^+^ permeation through a channel-forming venom component. The observation that crude venom is also able to induce currents when added to the intracellular milieu (Fig. 8d) suggests that a direct activation of Na^+^ channels following binding to an extracellular domain is not the case, and a pore-forming mechanism is more likely.

Scyphozoa venom is thermolabile^49^. Proteins are generally denaturated following exposure to high temperature and pore-forming toxins are proteinaceous macromolecules. *P. noctiluca* crude venom lost its current-inducing activity after boiling (Fig. 8b), which is in agreement with the hypothesis of a protein component having an essential role in eliciting the observed currents. Accordingly, exposure of venom to temperatures >40 °C significantly reduced its hemolytic activity^16^.

The plasma membrane of mammalian cells is highly permeable to water. Consequently, ion fluxes invariably lead to an osmotic-driven flux of water^29^. Based on this consideration, the venom-induced Na^+^ and Cl^−^ influx most likely generates an obligated influx of water, therefore explaining the abrogation of RVD and dramatic cell swelling in hypotonic solution (Fig. 1c) and cell swelling in isotonic solution (Fig. 1d). Venom-dependent enhancement of hypotonicity-induced cell swelling further activates IClswell (Figs 2 and 3), which relies on endogenous, swelling activated, NPPB-sensitive chloride channels (Fig. 3d). The link between the venom-induced current and cell swelling is strongly supported by the observation that conditions abrogating the venom-induced current (*i.e.* exposure of cells to the ion transport inhibitors amiloride and DIDS, Fig. 6c and d, and removal of Na^+^ and reduction of Cl^−^ concentration in the experimental solutions, Fig. 7d) also prevent the venom-induced increase in cellular volume (Fig. 7e). In addition, the observation that the relationship between venom dose and volume increase is linear (Fig. 1e), rather that sigmoidal and saturating, further substantiates the hypothesis of an involvement of a pore-forming component in determining the cell swelling, rather than the activation of endogenous conductances, that would most likely show a ceiling effect. Interestingly, venom-induced cell swelling was also observed in neuroblastoma cells exposed to equinatoxin II from the Antozoan *Actinia equina*, and was found to be dependent on the presence of Na^+^ and Ca^2+^ in the extracellular medium^50^.

The origin of the small Cl^−^ component of the venom-induced current observed in isotonic condition is less easily explainable and may be the consequence of activation of endogenous channels resistant to NPPB and DIDS or Cl^−^ permeation through a pore-forming component of crude venom.

To conclude, exposure to *P. noctiluca* crude venom causes to an electrogenic influx of ions, mainly Na^+^, through a permeation pathway excluding K^+^ and divalent cations and sensitive to the Na^+^ channel blocker amiloride. Influx of ions followed by an obligated, osmotically-driven influx of water may explain prominent venom-induced cell swelling and its cytolytic and hemolytic properties. This phenomenon is compatible with the activity of a pore-forming protein component. Whether treatment with amiloride or heat can help prevent or reduce pain and other consequences of the sting warrants further investigation.

## Materials and Methods

### Specimens collection

Specimens of *Pelagia noctiluca* (Cnidaria; Scyphozoa) were collected during the Spring-Summer 2014 in the Strait of Messina (Italy) and brought to the laboratory in tanks. Oral arms were immediately excised for nematocysts isolation and crude venom extraction.

### Nematocysts isolation and crude venom extraction

Nematocysts were isolated from *P. noctiluca* oral arms as previously described^51^. Shortly, after excision, the oral arms were submerged in distilled water for 2 h at 4 °C. In these conditions, undischarged nematocysts are released following osmotic lysis of nematocytes. After a complete detachment of the epidermis, the underlying tissue was removed from the suspension containing both the epidermis and nematocysts. Further release of nematocysts still attached to the epidermal tissue was induced by stirring. The nematocysts were repeatedly washed in distilled water and filtered through plankton nets (100, 60 and 40 μm mesh nets were used in this order) to remove most of the tissue debris, and then centrifuged at 4 °C (ALC PK 120 R centrifuge, 4000 *xg* for 5 min). The suspension was immediately used for venom extraction or, alternatively, frozen at −20 °C until use. The nematocysts obtained with this method were classified as holotrichous isorhizas, according to Mariscal^52^.

Crude venom was extracted from nematocysts as previously described^16^. Shortly, nematocysts were defrosted, filtered, washed in distilled water and re-suspended in 0.01 M phosphate buffer containing 0.9% NaCl (pH 7.4, osmotic pressure = 300 mOsm/Kg_H20_). Crude venom was extracted by sonication (Sonoplus, 70 mHz, 30 times, 20 s) on ice of a suspension of 90 nematocysts/μL. After sonication, the crude venom extract was separated from crushed capsules by centrifugation (4 °C, 4000 *xg*, 10 min), subjected to determination of protein content by BioRad Protein Assay (BioRad, Richmond, CA, USA), lyophilized and stored at −20 °C or −80 °C. Lyophilized venom was resuspended directly in the experimental solution (vehicle) before use in experiments.

### Cell lines

Human embryonic kidney (HEK) 293 Phoenix^53^ and HeLa (human cervical adenocarcinoma, American Type Cell Culture Collection CCL-2) cells were cultured in Minimum Essential Eagle Medium (Sigma-Aldrich, Austria) supplemented with 10% fetal bovine serum (Lonza), 2 mM L-glutamine, 100 U/ml penicillin, 100 μg/ml streptomycin and 1 mM pyruvic acid (sodium salt). NIH/3T3 cells (mouse embryonic fibroblasts, American Type Cell Culture Collection CRL-1658) were cultured in Dulbecco’s Modified Eagle’s Medium (Sigma-Aldrich, Austria) supplemented with 10% bovine calf serum (Lonza), 100 U/ml penicillin and 100 μg/ml streptomycin.

The cells were maintained at 37 °C, 5% CO_2_, 95% air and 100% humidity. Subcultures were routinely established every second to third day by seeding the cells into 100 mm diameter Petri dishes following trypsin/ethylenediaminetetraacetic acid (EDTA) treatment. For patch clamp experiments, cells were seeded on glass coverslips (diameter, 10 mm) contained in 30 mm diameter Petri dishes and grown overnight.

### Cell viability tests

To establish the maximal non-lytic dose of crude venom, cells were exposed for 15 min to an isotonic solution (solution 1, Table 1) containing increasing concentrations of crude venom and their integrity was assessed by the Trypan blue dye exclusion test. The non-lytic dose (no cell lysis was observed) of crude venom was 0.025 μg/μl for all cell types mentioned above. With venom concentrations >0.04 μg/μl, cell lysis was observed in most cells, thus preventing volume or current measurements.

### Cell volume measurements

Cell volume measurements were performed as previously described^54^. Shortly, HEK 293 Phoenix cells were grown on 18 × 18 mm coverslips successively placed upside down on a glass slide. A make-shift perfusion chamber was assembled by placing double sided tape between the glass slide and the coverslip with cells. The extracellular medium was completely and rapidly exchanged by adding the experimental solution to one side of the coverslip and removing it at the opposite side with strips of filter paper.

Cell volume measurements were performed on cells sequentially exposed to: isotonic solution (solution 1, Table 1) for 5 or 15 min; hypotonic solution (solution 2, Table 1; ~15% reduction of osmolality by omission of mannitol) for 30 min; isotonic solution for 5 min. Crude venom (0.025 μg/μl) or the vehicle were added to the isotonic or hypotonic solutions, as indicated. In addition, volume of cells was monitored in isotonic or isotonic Na^+^ -free/low Cl^−^ solution (solution 1 or 7, Table 1) containing 0.0025–0.04 μg/μl crude venom or the vehicle for 30 min.

Cell volume measurements were taken from cells demonstrating strong adhesion to the coverslip after checking with a microscope (Leica DMLS, 400 × magnification; Leica Microsystems GmbH, Wetzlar, Germany) during continuous perfusion. For each experiment, 30–65 images/cell (1 image/min) were recorded with a phase contrast microscope (Leica DMLS, 400 × magnification; Leica Microsystems GmbH) connected to a video camera (Digital CCD camera) and a computer equipped with suitable software (Movie Maker; Microsoft Co., Redmond, WA, USA). Cell diameter was measured for each recorded image and, assuming the cell as a sphere, cell volume was successively calculated and expressed as V/V_0_ or Vmax/V_0_, where V and V_0_ represent, respectively, the volume of a cell at a given time and the average of the volume of the same cell in isotonic solution. Vmax represents the maximal volume observed for a given cell over the experimental period.

### Patch clamp experiments

Single cells were selected by phase-contrast microscopy and voltage clamped using the whole-cell patch clamp technique as previously described^32^. Bath and pipette solutions (Tables 1 and 2) were designed to isolate either chloride, potassium or sodium currents. Crude venom (0.025 μg/μl) or the vehicle were added to the bath or pipette solutions, as indicated. The resistance of the glass pipettes filled with the pipette solution 1 (Table 2) and immersed in the bath solution 1 (Table 1) was 3 to 8 MΩ. Fast exchange of the bath solutions was accomplished using a perfusion system with a flow rate of 5 ml/min and a bath volume of ∼300 μl. The bath solutions were grounded with an Ag/AgCl electrode and a 4% agar/100 mM KCl bridge. For data acquisition, an EPC-10 (HEKA Elektronik, Germany) amplifier controlled by a Macintosh computer running the Patch Master (HEKA Elektronik, Germany) software was used. Access resistance as well as fast and slow capacitance were compensated and monitored throughout the recordings. All current measurements were filtered at 5 kHz and digitized at 50 kHz. The cells were held at 0 mV, and step pulses of 400 ms duration were applied from 0 mV to +40 mV every 20 s to monitor current changes over time. To establish the current-to-voltage (I/V) relationship, step pulses of 500ms duration were applied every 5 min from −120 mV to +100 mV in 20 mV increments from a holding potential of 0 mV. For K^+^ currents recordings, the holding potential was −60 mV. For data analysis, Fit Master (HEKA Elektronik, Germany) and Excel (Microsoft, USA) software were used. The current values (expressed as pA) were normalized to the membrane capacitance (expressed as pF) to obtain the current density (expressed as pA/pF), which gives a measure of the current magnitude independent from the cell size. To determine the % of current inhibition, current values were subtracted for the current of leakage. The current measured in isotonic bath solution 1 (Table 1) before the addition of crude venom was considered as current of leakage. Membrane potentials were corrected for liquid junction potentials. All experiments were carried out at room temperature.

### Salts and chemicals

All salts and chemicals used were of *per analysis* grade.

### Statistical Analysis

All data are expressed as arithmetic means ± S.E.M. For statistical analysis, GraphPad Prism software (version 4.00 for Windows, GraphPad Software, San Diego, California, USA) was used. Significant differences between data sets were tested by two-tailed, paired or unpaired Student’s t test or one or two-way analysis of variance (ANOVA) with Bonferroni’s post-hoc test, as appropriate. For calculation of Erev, the current-to-voltage relationships were fitted with second-order polynomials and Erev was determined by interpolation with the abscissa. Statistically significant differences were assumed at p < 0.05; (n) corresponds to the number of cells.

## Additional Information

**How to cite this article:** Morabito, R. *et al*. Crude venom from nematocysts of *Pelagia noctiluca* (Cnidaria: Scyphozoa) elicits a sodium conductance in the plasma membrane of mammalian cells. *Sci. Rep.* **7**, 41065; doi: 10.1038/srep41065 (2017).

**Publisher's note:** Springer Nature remains neutral with regard to jurisdictional claims in published maps and institutional affiliations.

## Supplementary Material

Supplementary Information

## Figures and Tables

**Table 1:** Composition (in mM) of extracellular (bath) solutions for whole-cell patch-clamp and cell volume regulation experiments.

	1 Isotonic, NaCl-rich	2 Hypotonic, NaCl^−^-rich	3 Isotonic K^+^-rich	4 Hypotonic K^+^-rich	5 Low Cl^−^	6 Na^+^-free	7 Na^+^-free, low Cl^−^
Na^+^	125	125	5.4	5.4	125		
K^+^			115	115			
NMDG^+^						125	125
Ca^2+^	2.5	2.5	1.8	1.8	2.5	2.5	2.5
Mg^2+^	2.5	2.5	0.5	0.5	2.5	2.5	2.5
Cl^−^	135	135	10	10	20	135	10
Gluconate			115	115	115		125
EGTA							
HEPES	10	10	5	5	10	10	10
Mannitol	50		70		50	50	as needed
Glucose			5	5			

Osmotic pressure was ~300–310 mOsm/Kg_H2O_, except for solutions 2 and 4, where mannitol was omitted and osmotic pressure was ~260 and 230 mOsm/Kg_H2O_, respectively; pH was 7.4. EGTA, ethylene glycol-bis (β-aminoethyl ether)-N,N,N′,N′-tetraacetic acid; HEPES, 4-(2-hydroxyethyl)-1-piperazineethanesulfonic acid; NMDG^+^, N-methyl-D-glucamine.

**Table 2:** Composition (in mM) of pipette filling (intracellular) solutions for whole-cell patch-clamp experiments.

	1 Cl^−^-rich	2 K^+^-rich	3 Low Cl^−^
**Cs**^**+**^	125		125
**K**^**+**^		115	
**Mg**^**2+**^	5	5	5
**Cl**^**−**^	135	10	10
**Aspartate**			125
**Gluconate**		115	
**EGTA**	11	5	11
**HEPES**	10	5	10
**ATP**	4	5	4

ATP was added to solutions as a salt of Mg^2+^, pH was 7.2, osmotic pressure was adjusted to ~300 mOsm/Kg_H2O_ with mannitol, as needed. EGTA, ethylene glycol-bis (β-aminoethyl ether)-N,N,N′,N′-tetraacetic acid; HEPES, 4-(2-hydroxyethyl)-1-piperazineethanesulfonic acid.
