# Supplementary material for: Crude venom from nematocysts of Pelagia noctiluca (Cnidaria: Scyphozoa) elicits a sodium conductance in the plasma membrane of mammalian cells
Source: Sci Rep. 2017 Jan 23;7:41065. doi: 10.1038/srep41065 (PMC5253680; doi:10.1038/srep41065)

Supplementary Information

Crude venom from nematocysts of *Pelagia noctiluca* (Cnidaria: Scyphozoa) elicits a sodium conductance in the plasma membrane of mammalian cells

Rossana Morabito[1], Roberta Costa[2], Valentina Rizzo[1], Alessia Remigante[1], Charity Nofziger[2], Giuseppa La Spada[1], Angela Marino[1], Markus Paulmichl[2*], and Silvia Dossena[2*]

[1]University of Messina, Department of Chemical, Biological, Pharmaceutical and Environmental Sciences, Viale Ferdinando Stagno D'Alcontres 31, Messina, I-98166, Italy

[2]Paracelsus Medical University, Institute of Pharmacology and Toxicology, Strubergasse 21, Salzburg, A-5020, Austria.

[*]silvia.dossena@pmu.ac.at; markus.paulmichl@pmu.ac.at

Supplementary Table 1. Effect of crude venom on the regulation of volume in HEK 293 Phoenix cells. Panels a, b and c refer to Fig. 1. ***: p<0.001 compared to the respective vehicle at the same time point, two-way ANOVA with Bonferroni's post-hoc test; n.s., §, §§, §§§: not significant, p<0.05, 0.01 and 0.001 compared to isotonic solution (0 min), one-way ANOVA with Bonferroni's post-hoc test.

		**V/V_0**		
	**n**	**Isotonic solution (0 min)**	**Hypotonic solution (6 min)**	**Hypotonic solution (30 min)**
**Vehicle, panels a and b**	4	1.000 ± 0.001	1.285 ± 0.034§§§	1.012 ± 0.013 n.s.
**Crude venom, panel a**	3	1.000 ± 0.060	1.218 ± 0.050§	1.380 ± 0.012***§§
**Crude venom, panel b**	3	1.000 ± 0.003	1.321 ± 0.029§§§	1.654 ± 0.0250***§§§
**Vehicle, panel c**	5	1.000 ± 0.003	1.197 ± 0.040§§	0.923 ±0.032 n.s.
**Crude venom, panel c**	5	1.335 ± 0.060***	1.568 ± 0.070***§	2.330 ±0.030***§§§

Legend to Supplementary Figures

Supplementary Fig. S1. Properties of voltage-dependent, potassium-selective channels in HEK 293 Phoenix. a, original current recordings and **b**, current density (pA/pF) to voltage (mV) relationship obtained in whole-cell configuration with pipette solution 2 (**Table 2**) and isotonic, K^+-rich bath solution 3 (**Table 1**), left, or after a 10 min exposure to a hypotonic, K^+-rich bath solution (solution 4, **Table 1**), right. *, **: $p<0.05$, $p<0.01$, paired Student's t test, n=6. No significant current decrease was observed in cells exposed for 10 min to the hypotonic solution, paired Student's t test, n=6.

Supplementary Fig. S2. The current-inducing activity of crude venom is not cell- an species-specific. Current density (pA/pF) to voltage (mV) (**a, b, d** and **e**) and current density (pA/pF) to time (sec) relationships (**c** and **f**) obtained from human HeLa (**a-c;** n=6) and mouse NIH/3T3 cells (**d-f;** n=5) in whole-cell configuration with pipette solution 1 (**Table 2**) and bath solution 1 (isotonic, **Table 1**), before (0 min) and after addition of the vehicle (**a, d**) or 0.025 µg/µl crude venom (**b, e**) to the bath solution. *: $p<0.05$, ** $p<0.01$ at all applied voltages except 0 mV compared to vehicle; in **c**, $p<0.05$ for time points >300 sec; in **f**, differences between data sets were not statistically significant; unpaired Student's t test.

Supplementary Fig. S1

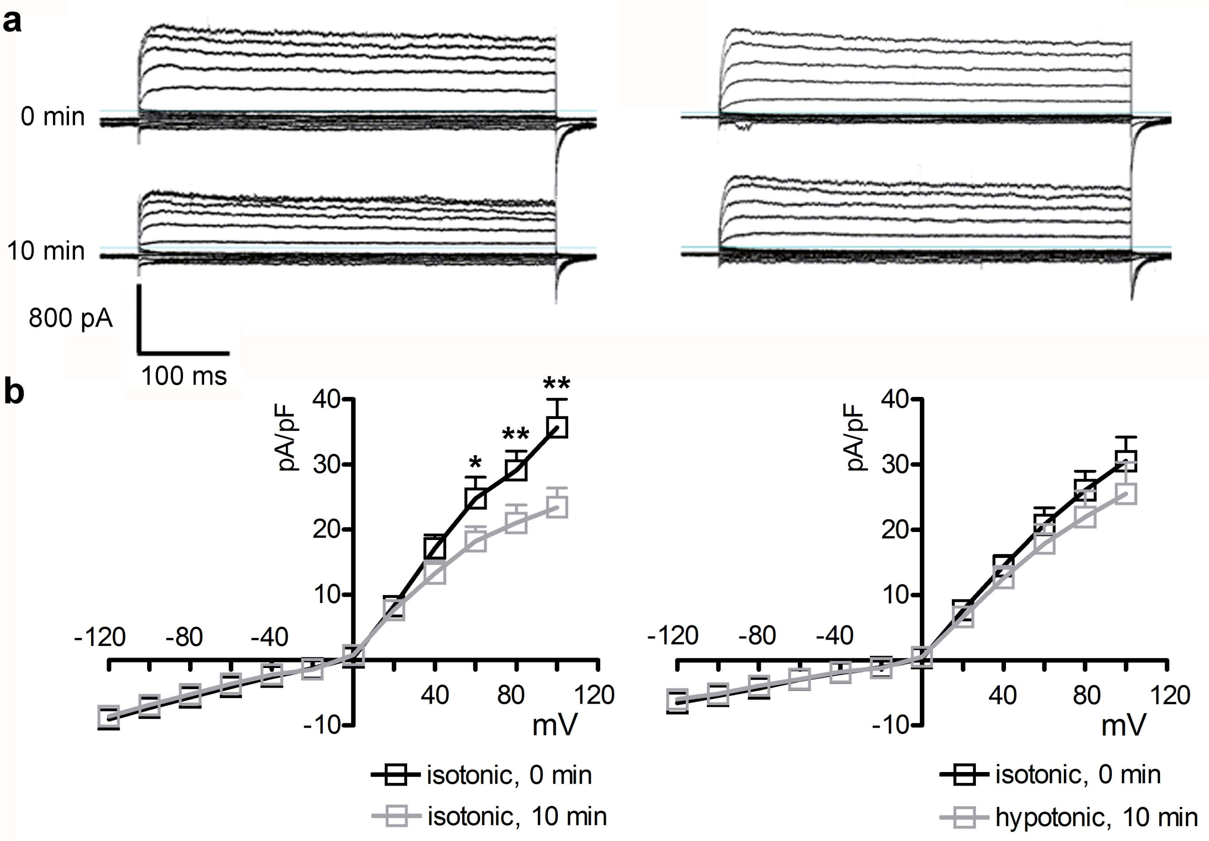

Supplementary Fig. S2

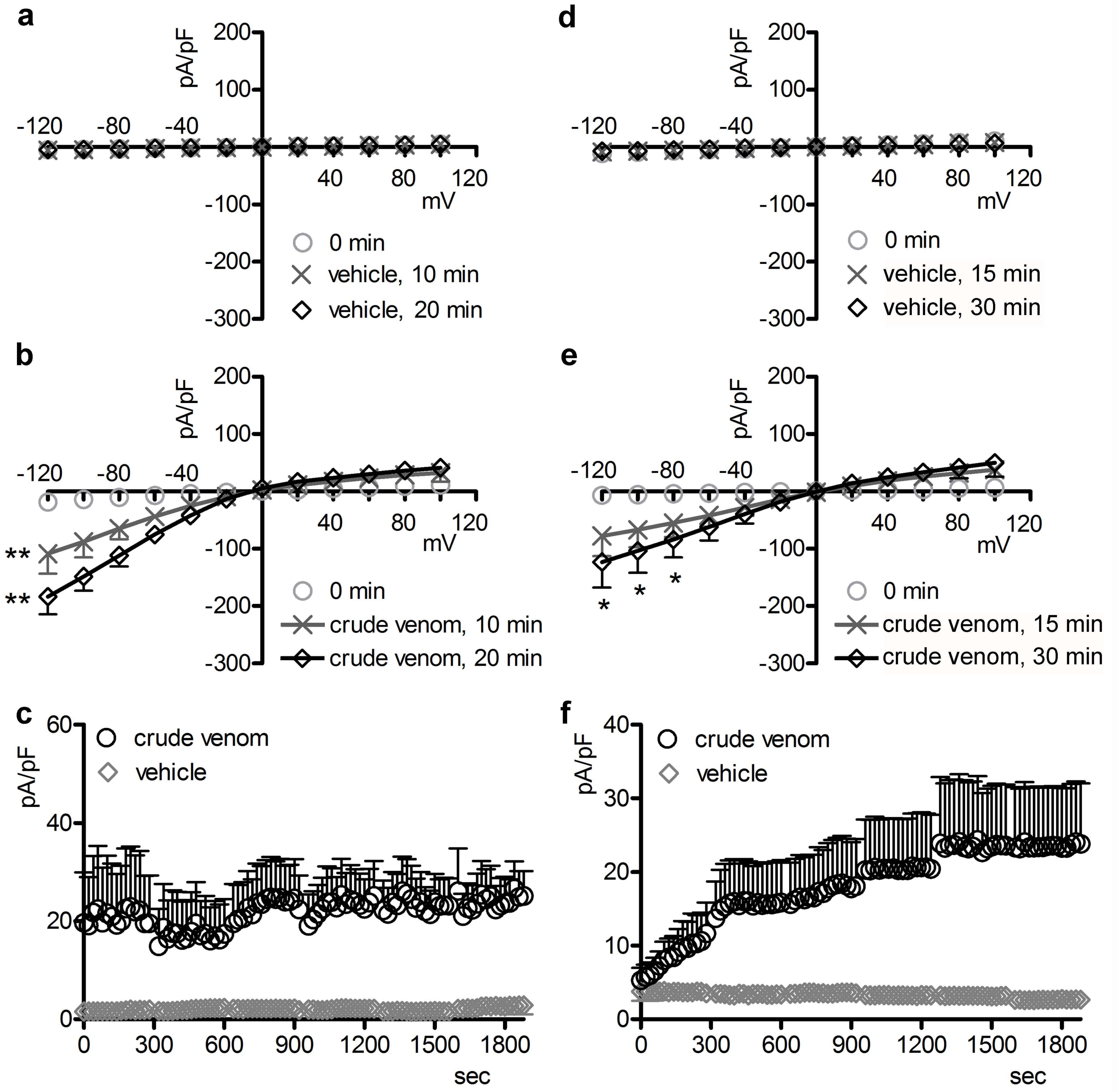

Supplement: Supplementary Information [file srep41065-s1.pdf]
